# Diagnostic accuracy of fetal MRI to detect cleft palate: a meta-analysis

**DOI:** 10.1007/s00431-019-03500-x

**Published:** 2019-12-03

**Authors:** Hanneke E. M. van der Hoek-Snieders, Antonius J. M. L. van den Heuvel, Harmieke van Os-Medendorp, Digna M. A. Kamalski

**Affiliations:** 1 Clinical Health Sciences, Faculty of Medicine, Utrecht University, Utrecht, The Netherlands; 2 Department of Dermatology/Allergology, University Medical Centre Utrecht, Utrecht, The Netherlands; 3 Department of Otorhinolaryngology Head and Neck Surgery, University Medical Centre Utrecht, Heidelberglaan 100, G05.129, 3584 CX Utrecht, The Netherlands

**Keywords:** MRI, Diagnostic accuracy, Cleft palate, Prenatal

## Abstract

This systematic review aims to determine the diagnostic accuracy of fetal MRI for detecting cleft palate in fetuses at risk for orofacial clefts. Pubmed, Embase, and CINAHL were searched systematically. A diagnostic study was included if it performed MRI (index test) and postnatal examination (reference test) in fetuses at risk for orofacial clefts. Methodological quality was assessed using the QUADAS-2. A meta-analysis was performed with a random-effects model, calculating the pooled sensitivity, specificity, and area under the curve. The search resulted in eight studies (334 fetuses) to be included: four prospective and four retrospective studies. The applicability concern was low. There was, however, a risk of selection and information bias. All studies showed that MRI well predicted the chance of cleft palate. The sensitivity results were homogeneous, but heterogeneity was assumed regarding the specificity estimate (Cochrane’s *Q* test: *p* = 0.00). The pooled sensitivity was 0.97 (95% CI 0.93–0.99); the pooled specificity was 0.94 (0.89–0.97). The area under the curve was 0.98 (95% CI 0.98–0.99).

*Conclusion*: This meta-analysis shows that MRI has an excellent sensitivity and good to excellent specificity for diagnosing cleft palate in fetuses at risk for orofacial clefts. Future research should assess applicability for clinical care.

**Table:** 

**What is Known:** • *Using ultrasound for prenatal detection of cleft palate leads to misdiagnosis frequently.* • *MRI could potentially improve the prenatal detection rate of cleft palate.*	
**What is New:** • *Eight studies describe the diagnostic accuracy of MRI for detecting cleft palate.* • *Combined results show excellent sensitivity and good to excellent specificity*.

**Electronic supplementary material:**

The online version of this article (10.1007/s00431-019-03500-x) contains supplementary material, which is available to authorized users.

## Introduction

Orofacial clefts are one of the most common facial malformations [14]. The prevalence of orofacial clefts differs from 10.2 per 10,000 in the United States and Western Europe to 20.0 per 10,000 in Japan [44]. This condition can be either a cleft lip (CL), a cleft palate (CP), or both CL and CP (CLP) [26]. Orofacial clefts often result in feeding problems and difficulties in speech [18]. Also, many children with CLP or CP have chromosomal abnormalities, with the highest prevalence in children with isolated CP [39]. Approximately 50% of the isolated CP’s are associated with other malformations, such as congenital heart defects or urinary tract defects [26].

Orofacial clefts are diagnosed by physical examination and visual inspection of the infant directly after birth [17]. To evaluate the palate, the mouth is inspected with a flashlight, while a spatula is used to depress the tongue.

Orofacial clefts are increasingly diagnosed prenatally during the ultrasound (US) fetal anomaly scan, often performed around the 20th week of the pregnancy [14]. After detecting abnormalities during this US screening, it is advised to refer the pregnant women with the fetus to a tertiary center, where the diagnosis is further explored [26]. Fetuses with a family risk factor for CL, CP, or CLP can be referred directly to these centers, since orofacial clefts are strongly genetically related [6].

Prenatal diagnosis provides opportunities for prenatal counselling and planning postnatal management [12, 34]. Prenatal counselling aims to help parents to cope with having a baby with a cleft and allows parents to make extra preparatory arrangements for the baby. Prenatal counselling also includes giving parents expert advice and support in the choice of the feeding method (breast milk or formula) allowing parents to make the feeding decision prior to the birth [13]. A study including 29 couples reported that 96% of the parents considered prenatal counselling as beneficial [38]. Early detection of orofacial clefts can also enable organization of the delivery in a tertiary center [15] and lead to fewer medical complications [20].

Unfortunately, many clefts are not detected during the US screening, since the detection rate differs from 9 to 50% and is further limited by factors such as maternal obesity [1, 25]. Specifically the palate can often not be adequately visualized, due to acoustic shadowing, which results in misdiagnoses of the palate [1, 7]. A systematic review investigated the diagnostic accuracy of second-trimester US in low- and high-risk populations [25]. A total of 27 articles were included, among which six studies with fetuses at high risk for orofacial clefts. Most studies reported detection rates between 9 and 50%, but results varied widely, depending on the population (high/low risk), type of cleft, and gestational age at the time of the scan. Detection rates were lower in the high-risk population: 0 to 44% of the orofacial clefts were detected prenatally using US in fetuses at risk. For isolated CP, the detection rate was 0% in this population. Assessing the palate during prenatal screening is essential in fetuses at risk for orofacial clefts, since CP has the highest association with feeding difficulties and other abnormalities [13]. The presence of a cleft palate can sometimes be used to rule out other diagnoses, such as a tumor, an encephalocele, or a vascular of lymphatic malformation [34]. Also, a recent systematic review investigated the prevalence of associated anomalies related to cleft category and concluded that prenatal counselling should be tailored to cleft category (CL, CP, or CLP) [26]. Without accurate information about the condition of the palate, this recommendation cannot be fulfilled.

Studies show magnetic resonance imaging (MRI) has potential to improve the prenatal detection rate of CP and should be considered as a complement to the current diagnostic procedure in fetuses at risk for a CP [31, 35]. Fetuses are at risk if the US screening shows abnormalities or if a family risk factor is present [2]. To evaluate the diagnostic value of MRI, information about the sensitivity and specificity of performing prenatal MRI for detecting CP is needed. A high sensitivity of the test is important to provide the prenatal counselling, including information about specific risks of CP. On the other hand, a high specificity prevents needless diagnoses, subsequent stress, and further medical investigations that could be a risk for the fetus. Namely, invasive genetic tests are indicated for CLP and CP with or without associated anomalies, but not for CL [26]. Therefore, the authors consider sensitivity and specificity of equal importance in the prenatal diagnosis of CP.

To decide if the prenatal diagnostic procedure for fetuses at risk for orofacial clefts should be extended by performing an MRI, information about its diagnostic value is needed. A few studies with relative small sample sizes were conducted to estimate the sensitivity and specificity of MRI for detecting CP [13, 27, 28]. Currently, no systematic review or meta-analysis has been conducted. Therefore, the diagnostic accuracy of MRI in detecting CP remains unknown. The aim of this meta-analysis is to establish the sensitivity and specificity of MRI in the prenatal diagnosis of cleft palate in fetuses at risk for orofacial clefts.

## Methods

### Design

To establish the diagnostic accuracy of MRI for the prenatal detection of CP, a meta-analysis was conducted according to the method of the Cochrane Handbook for Systematic Reviews and following the steps of the preferred reporting items for systematic reviews and meta-analyses (PRISMA) statement [16, 19].

### Information sources

The databases PubMed, Embase, and CINAHL were searched, because the subject of this meta-analysis is biomedical and related to the nursing field. Moreover, these databases all contain diagnostic studies, suitable for the research question. In case of good but insufficient data presented in the studies, authors were mailed to gain more specific results that could be used in the meta-analysis.

### Search method

Two reviewers (HH and AH) independently searched in the electronic databases on the 15th of June 2019. The search query combined the keywords fetuses, magnetic resonance imaging, cleft palate, and derivatives of these terms. The search strategy is provided in detail Appendix. The keywords sensitivity and specificity were not included in the search query, because consistent use of those terms in electronic databases is doubted and could consequently lead to missing relevant articles [23]. The references of all included studies were examined.

### Inclusion criteria

The inclusion criteria included specifications of the patient-group and results of prior testing, since test accuracy can vary due to these factors [23]. A paper was included if (1) it was a full-text article, (2) published in English or Dutch, (3) included fetal MRI as index test and postnatal diagnosis as reference standard performed by physical examination or autopsy, (4) described the outcome as type of orofacial cleft with at least a distinction in CL and CP, (5) was conducted in a population of fetuses at risk for orofacial clefts (due to a positive or unclear US screening or orofacial cleft in a first degree relative), and (6) sensitivity or specificity were mentioned or calculable from study results.

Exclusion criteria were formulated concerning the research design, number of participants, and purpose of the study. Studies with < 10 participants were excluded, because small sample sizes cause concern regarding the power and precision of studies [5]. Case reports, editorials, opinions, dissertations, intervention studies, and reviews were excluded as well as studies using fetal MRI for another purpose than diagnosing orofacial clefts. There was no restriction on the date of publication.

All included articles were also included in the meta-analysis, since the inclusion criteria were narrow regarding the population, index and reference test and calculability of sensitivity and specificity. A subgroup of the study sample was included if part of the sample did not meet the inclusion criteria.

### Study selection

After removing duplicates, the two reviewers (HH and AH) independently evaluated the eligibility of the selected studies from the literature in two phases. The first phase consisted of title and abstract screening and the second phase consisted of screening full-text articles. Disagreements were resolved in a consensus meeting.

### Data extraction

Data were extracted regarding study methodology, nation, patient population, inclusion and exclusion criteria, details about the performance of the index test, and details about the performance of the reference test. Outcome data on CP detected by MRI and the postnatal diagnosis were extracted in 2 × 2 contingency tables, describing true positive (TP), false positive (FP), false negative (FN), and true negative (TN) results. Data extraction was performed independently by the two reviewers (HH and AH). Discrepancies between the reviewers were resolved in a consensus meeting.

### Data synthesis and statistical analysis

The statistical analysis was performed using Review Manager 5.3 and Meta-Disc software version 1.4 [40, 46]. Sensitivity and specificity along with 95% confidence intervals (CIs) were calculated for each study and presented in forest plots.

To determine the appropriateness of statistical pooling of accuracy estimates, homogeneity needed to be assessed [46]. Homogeneity means that the variation in the estimates of the test accuracy from different studies can be explained by study sampling error alone [9]. For this meta-analysis, the homogeneity of the sensitivity and specificity of the included studies was assessed in three ways [46]. First, the accuracy estimates of the forest plots were inspected visually. Estimates that lie along a line corresponding to the pooled accuracy estimate indicate homogeneity. Large deviations from this line indicate possible heterogeneity. Second, the Cochrane’s *Q* test for heterogeneity was performed to evaluate if the differences across the studies are greater than expected by chance alone, using a chi-square (*χ*^2^) distribution and k-1 degrees of freedom. A *p* value < 0.05 suggests the presence of heterogeneity. Third, the inconsistency index I-squared was calculated to quantify the amount of heterogeneity. An I-square > 50% indicates heterogeneity. Heterogeneity can arise from differences in cut-off point for defining a positive or negative result: a threshold effect [32]. Assessing a threshold effect is not applicable for this meta-analysis, because the thresholds used in interpreting MRI are unquantifiable since they depend on interpretation. Furthermore, a summary receiver operating characteristics curve (sROC) was presented to visualize the test sensitivity against the test specificity. Also, 95% CI was given. The area under the curve (AUC) was calculated and interpreted as follows: 0.5–0.6 = fail, 0.6–0.7 = poor, 0.7–0.8 = fair, 0.8–0.9 = good, 0.9–1 = excellent [29].

Finally, a weighted, pooled estimate of sensitivity and specificity was calculated using the random-effects method [19]. The weighting of the estimate depends on the number of participants included in this meta-analysis and is adjusted for the extent of heterogeneity.

### Methodological quality

The methodological quality of the included studies was assessed with the Quality Assessment of Diagnostic Accuracy Studies-2 (QUADAS-2), as the subject of this meta-analysis is determining diagnostic accuracy [45]. This instrument distinguishes between bias and applicability and consists of four domains: patient selection, index test, reference standard, and flow and timing. For risk of bias questions, the answer yes indicates high risk of bias, no indicates low risk of bias and the answer is “unclear” if insufficient data is reported to permit a judgment. Concerns regarding the applicability of results were raised if the patient selection, use of the index test, or the use of the reference test did not fully agree with the inclusion criteria of this meta-analysis.

Overall quality scores were not indicated, because different shortcomings may lead to different magnitudes of bias and it is difficult to fairly weigh each quality item [23]. Quality assessment was performed independently by the two reviewers (HH and AH) and all disagreements were resolved by consensus through discussion. 

## Results

### Study selection

**Fig. 1:**
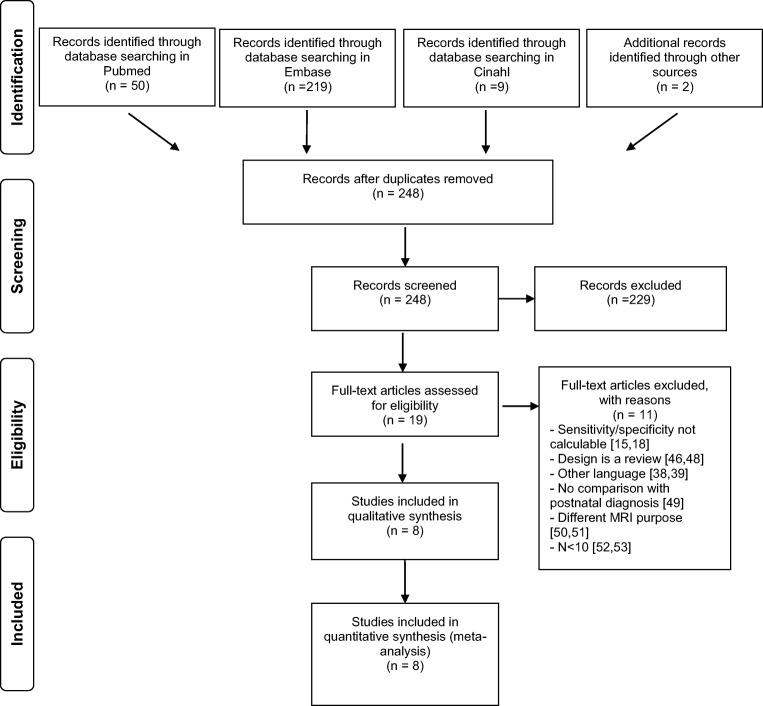
Flow scheme of included studies

The search strategy yielded a total of 246 unique records. Furthermore, two additional results were found with reference checking. After screening on title and abstract, 19 articles appeared to meet the inclusion criteria. After reading the full-text articles, 11 studies were eventually excluded, resulting in a total of eight definite inclusions with a combined total of 334 fetuses [3, 11, 13, 21, 27, 28, 43, 47]. The flow diagram displaying exact details is presented in Fig. 1.

### Study characteristics

Study characteristics are shown in Table 1. The studies are all conducted in different countries: five countries in Europe [3, 11, 13, 27], one in North America [21], and two in Asia [43, 47]. All studies were published in 2010 or later. Four studies had a prospective design [11, 13, 28, 43] and four studies had a retrospective design [3, 21, 27, 47]. The size of the study populations varied from 12 to 94 fetuses. The mean gestation time was 27.59 weeks at the time of the MRI. All studies included participants based on a positive US screening. Three studies also included fetuses based on a family risk factor: first degree family member having CL, CP, or CLP [11, 13, 43]. Two studies excluded fetuses with associated abnormalities, such as agenesis of corpus callosum (a brain abnormality) [11, 28]. Seven studies used a 1.5-T MRI system as the index test of different brands. In one study, the type of MRI scanner was not specified [21]. Also, all studies used the postnatal diagnosis as the reference test performed by physical examination or autopsy. Autopsy was used in case of fetal death or termination of pregnancy.

**Table 1:** Characteristics of included studies

First author	Year of publication	Nation	Design	No. of fetuses	Risk factor determined by	Exclusion criteria	Gestational time in weeks	Index test	Reference test
Bekiesinska-Figatowska	2014	Poland	Retrospective	62	Positive US diagnosis	Not mentioned	Not mentioned	1.5 Tesla MRI	Postnatal findings
Dabadie	2016	France	Prospective	22	Positive US diagnosis or family risk factor	Associated abnormalities	29.5 (27–34)	1.5 Tesla MRI	Postnatal findings
Descamps	2010	United Kingdom	Prospective	49	Positive US diagnosis or family risk factor	Not mentioned	34.4 (24–37)	1.5 Tesla MRI	Postnatal findings
Laifer-Narin	2019	United States	Retrospective	61*	Positive US diagnosis	No reference test available	26.4 (18–38)	Not mentioned	Postnatal- or autopsy findings
Mailath-Pokorny	2010	Austria	Retrospective	34	Positive US diagnosis	Not mentioned	26.0 (19–34)	1.5 Tesla MRI	Postnatal- or autopsy findings
Manganaro	2011	Italy	Prospective	27**	Positive US diagnosis	Associated anomalies	23.7 (19–33)	1.5 Tesla MRI	Postnatal- or autopsy findings
Wang	2011	Japan	Prospective	12	Positive US diagnosis or family risk factor	Not mentioned	28.0 (21–34)	1.5 Tesla MRI	Postnatal- or autopsy findings
Zheng	2019	China	Retrospective	94***	Positive US diagnosis	Not mentioned	26.1 (19–38)	1.5 Tesla MRI	Postnatal- or autopsy findings

No, number; NA, not applicable; US, ultrasound; MRI, magnetic resonance imaging

*19/61 Fetuses were lost to follow up or underwent termination of pregnancy without autopsy and not included in the meta-analysis

**2/27 Fetuses in twin pregnancy were not at risk for CP and not included in the meta-analysis

***6/94 Fetuses were lost to follow-up and not included in the meta-analysis

### Methodological quality

The risk of bias and applicability concerns are summarized in Table 2. The patient selection of four studies was judged as high risk of bias, since a case control design was not avoided and it was unclear whether a consecutive sample of patients was enrolled [3, 21, 27, 47]. In three studies, it was unclear how the participants enrolled [13, 28, 43]. One study clearly stated that all patients with CL, CLP, or CP were approached and included in the study after referral to two centers for further diagnostics [11].

**Table 2:** Summary of quality assessment using QUADAS-2

Study	Risk of bias				Applicability concerns		
	Patient selection	Index test	Reference standard	Flow and timing	Patient selection	Index test	Reference standard
Bekiesinska-Figatowska 2014	-	?	?	?	+	+	+
Dabadie 2016	+	?	?	+	+	+	+
Descamps 2010	?	?	?	+	+	+	+
Laifer-Narin 2019	-	?	?	-	+	+	+
Mailath-Pokorny 2010	-	-	-	+	+	+	+
Manganaro 2011	?	?	?	+	+	+	+
Wang 2011	?	-	-	+	+	+	+
Zheng 2019	-	?	?	-	+	+	+

+, low risk; -, high risk; ?, unclear risk

Two studies described a blinded procedure for interpretation of the index test [11, 13]. The other studies were unclear about this procedure [1, 3, 21, 27, 43, 47]. Although technical specifications of the MRI scanner were frequently provided, the radiologists criteria for diagnosing CP were unclear in all studies.

Regarding the applicability of the studies, all studies had the main aim of determining the diagnostic accuracy of the MRI in detecting CP or CLP [3, 11, 13, 21, 27, 28, 43, 47]. All studies used postnatal examination as the reference test, by physical examination or by autopsy. One study did not report about using a reference standard, but the affirmation with the postnatal diagnosis was confirmed after contact with the author [3].

### Results of individual studies

**Fig. 2:**
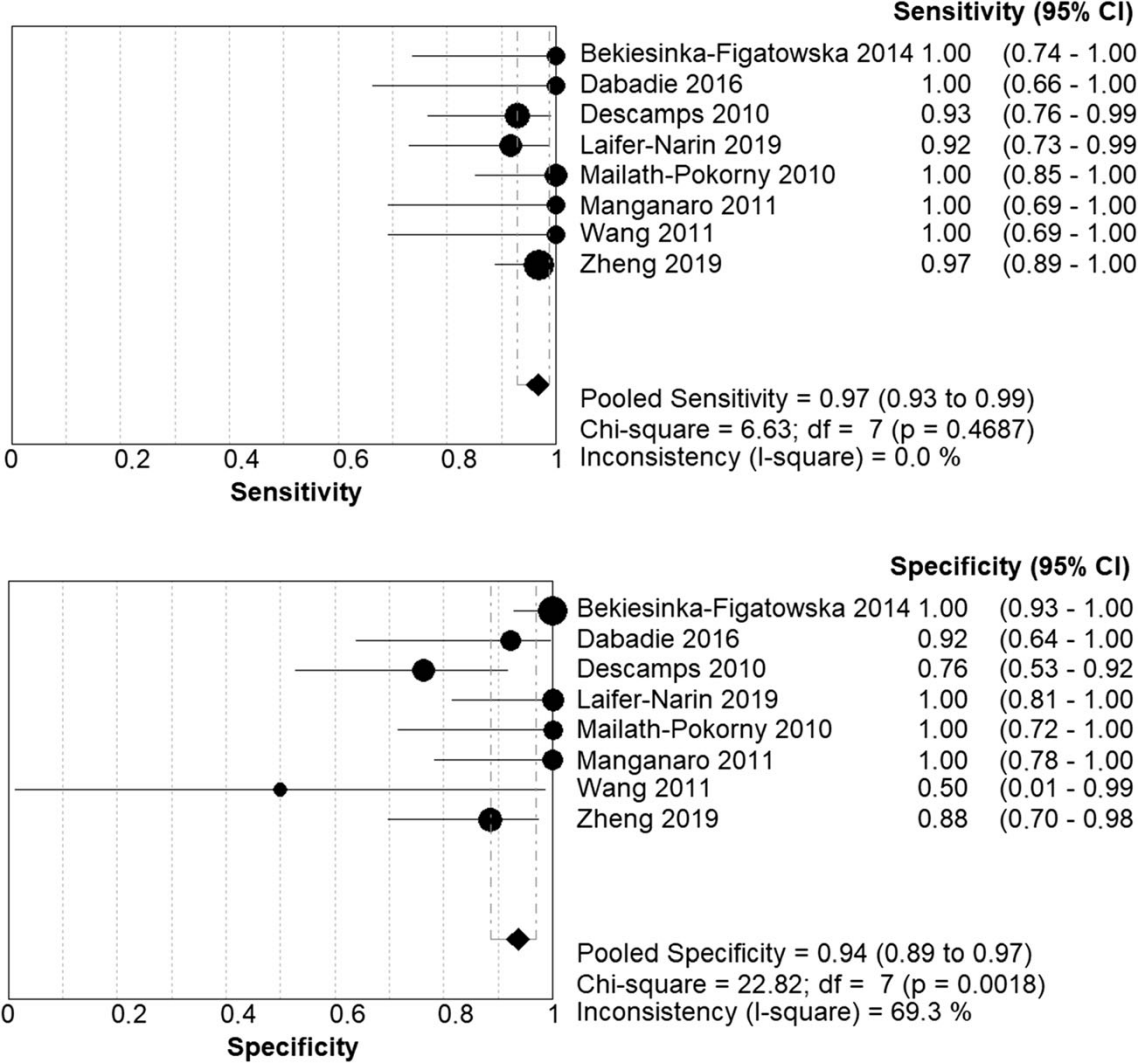
Forest plot of all included studies. CI: confidence interval; TP: true positive; FP: false posittive; FN: false negative; TN: true negative

The diagnostic accuracy of MRI in detecting CP is evaluated in this study in terms of the sensitivity and specificity. The results are visualized in Fig. 2 and Table 3.

**Table 3:** Distribution of positive and negative diagnoses per study and whether these were predicted correctly with MRI

	True positive	False positive	False negative	True negative
Bekiesinska-Figatowska 2014	12	0	0	50
Dabadie 2016	9	1	0	12
Descamps 2010	26	5	2	16
Laifer-Narin 2019	22	0	2	18
Mailath-Pokorny 2010	23	0	0	11
Manganaro 2011	10	0	0	15
Wang 2011	10	1	0	1
Zheng 2019	60	3	2	23

A combined total of 6 false negative diagnoses were reported in three studies [13, 21, 47] resulting in a sensitivity of respectively 0.93, 0.97, and 0.92 [13, 21, 47]. The other five studies had a sensitivity of 1.00 [3, 11, 27, 28, 43]. All sensitivity confidence intervals were in the range from 0.66 to 1.00.

A total of 10 fetuses received a false positive diagnosis. These diagnoses were made in four different studies and resulted in a specificity of respectively 0.76, 0.50, 0.92, and 0.88 [11, 13, 43, 47]. The other studies reported no false positive results, corresponding to a specificity of 1.00 [3, 21, 27, 28]. The specificity confidence intervals ranged from 0.01 to 1.00. The lowest specificity was reported in a study that included two negative cases, from which one case was correctly predicted [43].

### Homogeneity

The tabular results of the homogeneity analysis are included in Fig. 2. The forest plot shows that the estimates of the sensitivity lie roughly along the line of the pooled sensitivity (0.966). The Cochrane’s *Q* test statistic is 6.63, df = 7, *p* = 0.47, and the I-square is 0.0%. All three results indicate homogeneity.

For specificity, the forest plot shows more variation. Not all estimates lie along the line of the pooled specificity (0.94). The Cochrane’s *Q* test statistic is 22.82, df = 7, *p* = 0.00, and the I-square is 69.3%. All specificity results indicate heterogeneity.

### Meta-analysis

**Fig. 3:**
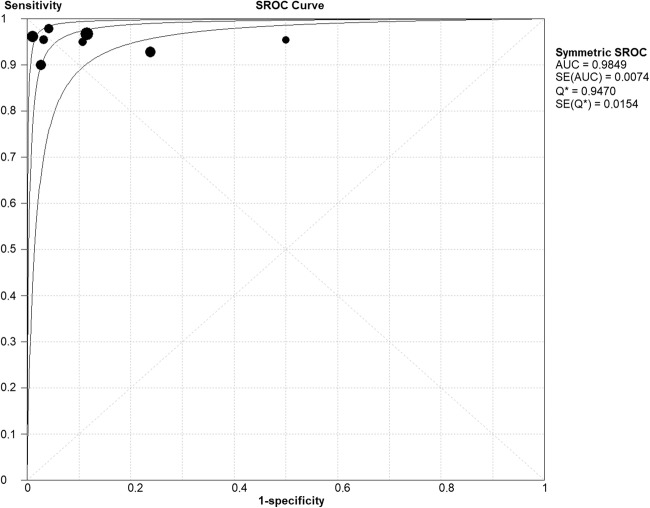
Summary receiver operating characteristic plot of all included studies. AUC: area under the curve; SE: standard error; Q: *Q*-statistic

The weighted pooled sensitivity from all eight studies was 0.97 (95% CI 0.928–0.988) and is interpreted as excellent. The weighted pooled specificity was 0.94 (0.89–0.97) and is interpreted as good to excellent. The sROC is provided in Fig. 3. The AUC is 0.98 (95% CI 0.98–0.99). This is interpreted as excellent.

## Discussion

This meta-analysis brings together all available evidence about the diagnostic accuracy of MRI for the prenatal diagnosis of CP. Combining the results of eight studies enabled to more precisely estimate the diagnostic accuracy of MRI, revealing excellent sensitivity and good to excellent specificity.

The methodological quality of this study is strengthened by the fact that the data collection, extraction, and synthesis were performed separately by two researchers. Also, the broad inclusion criteria used in this study, without additional search filters, contribute to the discovery of almost all available studies [23]. Furthermore, the power and precision of this study were increased, because a meta-analysis was performed [10]. However, some limitations are worth noting.

The reliability of this study was limited, because a small number of studies was included of which most included a relatively small sample size. Also, this meta-analysis does not provide information for subgroup analysis within the selected population. For instance, comparison could not be made between the diagnostic accuracy of isolated CP with CLP, because isolated CP was described in only five fetuses [3, 11, 27]. One study did not provide information about the total number of isolated CP and CLP diagnosis [13]. Consequently, the false negative diagnosis and two false positive diagnosis that occurred in this study could not be further analyzed. The other two false positive diagnosis occurred two fetuses with CLP [11, 43]. The impact of gestational age could also not be explored, because it was not mentioned in one study [3].

Furthermore, selection bias might have been caused by the language filter that was used. The search strategy revealed two studies in other languages, one in Czech and one in Chinese [4, 42]. The language criterion might have caused even more selection bias if other studies did not publish an abstract in English. Additionally, information bias might be present, since the MRI specifications have not been included in the meta-analysis. Although all studies use a 1.5-Tesla MRI, the resolutions of these MRI’s differ. The MRI resolution is likely to influence the diagnostic accuracy, but future research should confirm if a higher resolution contributes to a more accurate diagnosis.

Another limitation concerns the nature of the heterogeneity. Although the extent of heterogeneity was investigated thoroughly in this study, an assessment of the nature of the heterogeneity was not included in this meta-analysis for two reasons. First, a threshold effect, the primary cause for heterogeneity in diagnostic studies cannot be assessed, because the threshold in MRI assessment is implicit [23, 46]. It is therefore unclear to what extent study variation is caused by the application of different thresholds. Second, insufficient information is available about population variables and study characteristics to perform meta-regression. For example, the time of the physical examination could have influenced the performance of the reference test and consequently could have caused variation in the estimated diagnostic accuracy [17]. Since the included studies did not explicitly state the time of the physical examination, this variable could not be included in a meta-regression.

Further, the reliability of the reference test can be discussed [11, 17, 22, 37]. For diagnostic studies, it is crucial to have a reference test that accurately determines the presence or absence of the target condition [37]. Postnatal diagnosis is considered the reference standard in diagnosing CP [11, 41]. This is performed by thorough physical examination or autopsy. However, some requirements are needed to rely on this diagnosis. The World Health Organization states the importance of visual inspection with a light and with a tongue depressor as part of this physical examination [17]. In daily practice, if this recommendation is not satisfied, delayed diagnosis of CP is not uncommon [41]. The studies included in this meta-analysis do not describe the way of performing the postnatal diagnosis and the value of the reference standard could be cause for discussion. Additionally, it is suggested that by gaining more experience with interpreting MRI, interpretation skills significantly improve [13]. It is unclear to what extent a difference in experience was present in the included studies, and if this difference has led to variation between the studies.

After determining the diagnostic accuracy of a test, the applicability needs to be investigated [23]. This includes assessment of the safety of the test, the availability of materials and expertise, the increased burden of care and cost-effectiveness. First, the increased use of MRI in prenatal diagnoses warrants questions about the safety of this diagnostic instrument, including the possibility of developmental defects due to the exposure to the magnetic radiation [22]. Two reviews show no harmful effects of MRI performed at 1.5 Tesla and lower [24, 35]. A 10-year follow-up study including was cited [33]. This study included 74 pregnant woman and did not reveal significant differences in birth weight between children that were exposed and not exposed to prenatal MRI. However, to our knowledge, the follow-up results have not been published yet. Visualization of fetal structures is more difficult and involves more risks in the first trimester of the pregnancy [8, 24]. It is advised to avoid performing MRI before 13 weeks gestational age [36]. The studies included in this meta-analysis performed MRI between 18 and 38 weeks of gestational age, and therefore meet this criterion. Second, performing prenatal MRI requires specific material and expertise, such as knowledge about MRI safety and how to optimize fetal images [24]. Prenatal MRI is advised therefore to perform in multidisciplinary centers with fetal MRI expertise [24]. Consequently, the mothers of fetuses at risk for CP will generally need to travel further, to visit a center where the MRI can be performed. Also, the length and costs of the hospital visit will be increased due to this additional diagnostic test. By assessing the applicability of MRI in prenatal diagnoses of CP, alternative options should also be considered.

Just as interest grows in diagnosing CP using prenatal MRI, improving prenatal diagnosis of CP through US techniques also receives attention [15, 30]. For example, using clinical markers derived from 2D- and 3D sonography is suggested to improve correct diagnosis of CP in the first trimester [30] and a scoring system has been developed to help sonographers to correctly identify CP [15]. To summary all US innovations is beyond the scope of this study, but is recommended to be further explored in future research. Cost-effective studies including factors such as availability of equipment and skills are required to determine if MRI should be implemented in the diagnostic process of CP. It is recommended to include subgroups of fetuses in the decision-making process of diagnostic instruments for standard care, such as fetuses with a family risk factor of CP, fetuses with positive US diagnosis, fetuses with or without cleft lip, and fetuses with or without other anomalies. It is therefore not yet recommended to include MRI in standard prenatal care, but MRI could be used in specific cases relying on correct diagnosis.

In conclusion, this meta-analysis shows that MRI is an instrument with excellent sensitivity and good to excellent specificity for diagnosing CP in fetuses at risk for orofacial clefts. Findings indicate that the beneficial effect of performing MRI in this population should not be underestimated, since it provides opportunities to optimize prenatal management and counselling and planning of delivery and postnatal management. Additional research of good methodological quality should assess applicability for clinical care.

## Electronic supplementary material

ESM 1 (DOCX 14 kb)

## Abbreviations

AUC: Area under the curve
CI: Confidence interval
CL: Cleft lip
CLP: Cleft lip and palate
CP: Cleft palate
FP: False positive
FN: False negative
MRI: Magnetic resonance imaging
PRISMA: Preferred reporting items for systematic reviews and meta-analyses
QUADAS-2: Quality Assessment of Diagnostic Accuracy Studies-2
sROC: Summary receiver operating characteristics curve
TP: True positive
TN: True negative
US: Ultrasound
