# Supplementary material for: Diagnostic accuracy of fetal MRI to detect cleft palate: a meta-analysis
Source: Eur J Pediatr. 2019 Dec 3;179(1):29–38. doi: 10.1007/s00431-019-03500-x (PMC6942582; doi:10.1007/s00431-019-03500-x)
Supplement: Supplementary file 1 — (DOCX 14 kb) [file 431_2019_3500_MOESM1_ESM.docx]

| Database | Search string |
| --- | --- |
| Pubmed | (fetus[MeSH Terms]) OR fetus*[Title/Abstract]) OR fetal structure*[Title/Abstract]) OR fetal tissue*[Title/Abstract]) OR Pregnant Woman[Title/Abstract]) OR Woman, pregnant[Title/Abstract])))) AND ((((((((((cleft palate[MeSH Terms]) OR cleft palate*[Title/Abstract]) OR CL+P[Title/Abstract]) OR CP[Title/Abstract]) OR Orofacial cleft*[Title/Abstract]) OR Posterior palate*[Title/Abstract]) OR Cleft lip with cleft palate[Title/Abstract]) OR palate, cleft[Title/Abstract]) OR palates, cleft[Title/Abstract]) OR facial cleft*[Title/Abstract])))) AND (((((((((((((((((((((((((((((magnetic resonance imaging[MeSH Terms]) OR magnetic resonance imaging[Title/Abstract]) OR MRI[Title/Abstract]) OR MR imaging[Title/Abstract]) OR Imaging, Magnetic Resonance[Title/Abstract]) OR NMR Imaging[Title/Abstract]) OR Imaging, NMR[Title/Abstract]) OR Tomography, NMR[Title/Abstract]) OR Tomography, MR[Title/Abstract]) OR MR Tomography[Title/Abstract]) OR NMR Tomography[Title/Abstract]) OR Spin Echo Imaging[Title/Abstract]) OR Zeugmatography[Title/Abstract]) OR Imaging, Chemical Shift[Title/Abstract]) OR Chemical Shift Imagings[Title/Abstract]) OR Imagings, Chemical Shift[Title/Abstract]) OR Shift Imaging, Chemical[Title/Abstract]) OR Shift Imagings, Chemical[Title/Abstract]) OR Chemical Shift Imaging[Title/Abstract]) OR Tomography, Proton Spin[Title/Abstract]) OR Proton Spin Tomography[Title/Abstract]) OR Magnetization Transfer Contrast Imaging[Title/Abstract]) OR Echo Imagings, Spin[Title/Abstract]) OR fMRI[Title/Abstract]) OR Echo Imaging, Spin[Title/Abstract]) OR Imagings, Spin Echo[Title/Abstract]) OR Spin Echo Imagings[Title/Abstract]) OR prenatal imaging[Title/Abstract]) |
| Embase | fetus'/exp OR 'fetus' OR 'pregnant woman'/exp OR 'pregnant woman' OR 'fetal structure' OR 'fetal structures' OR 'fetal tissue'/exp OR 'fetal tissue' AND 'cleft lip face palate'/exp OR 'cleft lip face palate' OR 'cleft palate'/exp OR 'cleft palate' OR 'cleft face'/exp OR 'cleft face' OR cl+p OR cp OR 'orofacial cleft'/exp OR 'orofacial cleft' OR 'posterior palate' OR 'facial cleft'/exp OR 'facial cleft' AND 'nuclear magnetic resonance'/exp OR 'nuclear magnetic resonance' OR 'functional magnetic resonance imaging'/exp OR 'functional magnetic resonance imaging' OR 'mri'/exp OR mri OR 'magnetic resonance imaging'/exp OR 'magnetic resonance imaging' OR 'mr imaging'/exp OR 'mr imaging' OR 'imaging, magnetic resonance' OR 'nmr imaging'/exp OR 'nmr imaging' OR 'imaging, nmr' OR 'tomography, nmr' OR 'tomography, mr' OR 'mr tomography' OR 'nmr tomography' OR 'spin echo imaging'/exp OR 'spin echo imaging' OR 'zeugmatography' OR 'imaging, chemical shift' OR 'chemical shift imaging'/exp OR 'chemical shift imaging' OR 'proton spin tomography' OR 'magnetization transfer contrast imaging' OR 'fmri'/exp OR 'fmri' OR 'prenatal imaging' OR 'spin echo imaging'/exp OR 'spin echo imaging' |
| CINAHL | MH "Magnetic Resonance Imaging" OR TI ( "Magnetic Resonance Imaging" OR MRI OR "MR imaging" OR "Imaging Magnetic Resonance" OR "NMR Imaging" OR Tomography OR "Spin echo imaging" OR Zeugmatography OR "Chemical shift imaging" OR "Magnetization Transfer Contrast Imaging" OR "Spin Echo Imagings" OR fMRI OR "prenatal Imaging" ) OR MH Tomography OR AB ( "Magnetic Resonance Imaging" OR MRI OR "MR imaging" OR "Imaging Magnetic Resonance" OR "NMR Imaging" OR Tomography OR "Spin echo imaging" OR Zeugmatography OR "Chemical shift imaging" OR "Magnetization Transfer Contrast Imaging" OR "Spin Echo Imagings" OR fMRI OR "prenatal Imaging" ) |

**Appendix 1: Search string**
